# Notes of Clinical Practice. Staphyloraphy. Necrosis of Inferior Maxillary

**Published:** 1869-02

**Authors:** Wm. Mason Turner

**Affiliations:** Surgery. Service of Prof. S. D. Gross.


### ARTICLE VII.

### *Notes of Clinical Practice. Staphyloraphy. Necrosis of Inferior Maxillary.*

By Dr. Wm. Mason Turner.

(Surgery. Service of Prof. S. D. Gross.)

Case I.—A young man, nineteen or twenty years of age, hailing from Ohio, came here to consult the Professor. On examination, there was found a *staphyloraphy*—ordinarily known as fissured, or cleft palate. It involved only the soft parts, and was congenital. Prof. Gross had operated on the patient a few days before he had been presented to the class. The inflammation following the operation was not more than was expected, and what was, in fact, necessary to promote perfect union. The operation performed, consisted in paring the edges of the cleft,—bringing the raw surfaces together and retaining them *in situ*, with twisted sutures, held firmly by compressed shot. Between these four or five sutures, a single *interrupted* suture was introduced. This latter was removed in five days, the others being allowed to remain in until the eighth day. A solution of nitrate of silver (ten grains to the fluid ounce), had been applied; but in case there was a further demand for stimulation of the parts, the solid stick would be cautiously used. Good food, and general outdoor exercise were now demanded. *Immediate* improvement in speech could not be expected, but it would improve by constant practice, especially the practice of pronouncing the letters of the alphabet. The operation, however, was a success beyond a peradventure.

Case II.—A lad of thirteen years, a healthy-looking fellow. Prof. Gross saw him in the summer of 1867. History of the case in brief: since the boy had a tooth extracted, had suffered with his lower jaw. The Professor remarked, that he surmised, that the alveolar process had been broken from the rudeness of the dental operation. At all events, *necrosis* of the *lower maxilla* had followed. At the time the lad first presented himself, the parts were scraped well, and a small quantity of dead bone removed, but the improvement was only temporary—an abscess had formed, and at last the boy came back again. An operation was determined upon. After anæsthesia had been induced, a probe was introduced through the outside fistulous opening, into the wound, and the surface of the maxilla, very much roughened, was detected. The parts were next laid open by a free incision, and quite a large piece of dead bone taken out. Every thing of a semi-organized, granulous and hurtful growth, was completely cleaned away—the Professor laying great stress on this precaution. Warm solutions of potass. permang. or liq. sod. chlorinatæ, were directed.—*New Orleans Journal of Medicine.*

